# Orthopedic Manifestations of Mobius Syndrome: Case Series and Survey Study

**DOI:** 10.1155/2016/9736723

**Published:** 2016-02-08

**Authors:** Philip McClure, David Booy, Julia Katarincic, Craig Eberson

**Affiliations:** ^1^Warren Alpert Medical School, Brown University, Providence, RI 02905, USA; ^2^Pediatric Hand Surgery, Rhode Island Hospital, Providence, RI 02905, USA; ^3^Division of Pediatric Orthopedics, Rhode Island Hospital, Providence, RI 02905, USA

## Abstract

*Background*. Mobius Syndrome is a rare disease defined by bilateral congenital 7th nerve palsy. We focus on reporting the prevalence of orthopedic disease in this population.* Methods*. Twenty-three individuals with Mobius Syndrome underwent orthopedic physical examination, and additional 96 patients filled out a survey for self-reported orthopedic diagnoses.* Results*. Clubfoot was present in 60% of individuals in the physical exam series and 42% of those in the survey. Scoliosis was present in 26% and 28%, respectively. Poland's Syndrome was present in 17% and 30%. In addition to these findings, 27% of patients reported having difficulty with anesthesia, including difficulty in intubation and airway problems.* Conclusion*. An increased prevalence of scoliosis, clubfoot, transverse limb deficiencies, and Poland's Syndrome is identified in the setting of Mobius Syndrome. In the setting of several deformities often requiring surgical correction, a high incidence of anesthetic difficulty is noted and should be discussed with patients and other providers during surgical planning.

## 1. Introduction

Mobius Syndrome is a rare syndrome with undefined incidence in the United States. In 2003, an estimated 4 out of 189,000 births in Holland were affected [1]. Originally described in 1880 and subsequently named in 1888 after Dr. Paul Mobius described the first case series, Mobius Syndrome has been the subject of continued medical research; a great deal of progress has been made. Congenital facial diplegia and cranial nerve six palsy are the hallmark of Mobius Syndrome. Limb anomalies may also be present [2]. The initial presentation is often for breastfeeding difficulty [3, 4].

Clubfoot has been identified as the most common orthopedic association of Mobius Syndrome; congenital hand differences and Poland's Syndrome are also present in higher frequency. Facioscapulohumeral muscular dystrophy has been associated with Mobius Syndrome and is a progressive proximal muscle disease leading to progressive weakness about the hip and shoulder [5, 6]. An association with Klippel-Feil, or congenital fusion of cervical vertebrae, has also been identified [7, 8].

A consistent etiology for the condition has not been definitively identified; however, multiple contributing factors have been described in the literature, including chromosomal abnormalities secondary to teratogenic exposure [1, 9, 10]. One prevailing hypothesis includes a vascular disruption to the subclavian artery in the sixth week of embryogenesis leading to decreased arterial supply to the brain stem, which may also lead to spine and extremity manifestations. The increasing incidence of Mobius Syndrome on the trials of misoprostol use appears to support this theory [7, 10, 11].

Familial patterns have also been identified but do not appear to contribute to a majority of cases [12]. Some have suggested that the presence or absence of limb anomalies may be an important indicator for decreased or increased risk of inheritance, respectively [3]. Various modes of inheritance have been identified across the literature of multiple subspecialties [12–15]. A specific and consistent genetic abnormality has not been clearly identified, though efforts continue and have shown some variation [16–22]. The authors postulated that identifying common orthopedic manifestations of this syndrome would assist physicians who care for affected patients to promptly establish a diagnosis and treatment plan.

## 2. Methods

In an effort to improve the understanding of the orthopedic associations of Mobius Syndrome, we distributed a survey to the membership of the Mobius Syndrome Foundation after IRB approval had been obtained. The survey included questions regarding the upper extremity, lower extremity, and spine as well as anesthetic and airway concerns. The survey was distributed through the Mobius Syndrome Foundation Newsletter and social media site, and anonymous responses were collected through an established survey website.

Survey responses were tabulated in spreadsheet format and analyzed. Questions were designed as yes and no answers, with an option to further describe or clarify responses as individuals saw fit.

In addition, 23 individuals with Mobius Syndrome were interviewed and examined by the primary author at the Mobius Syndrome Foundation meeting in July 2014. Each patient received a full history, orthopedic exam, and review of any available imaging.

## 3. Patient Series

**Figure 1:**
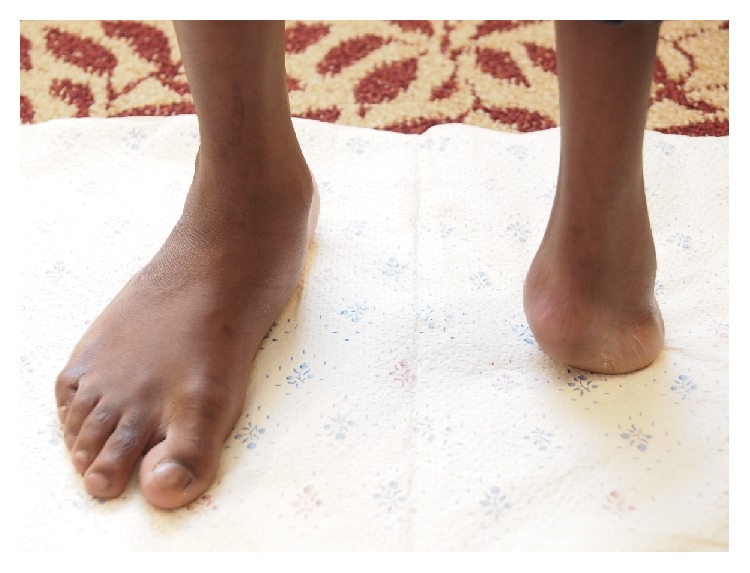
Transverse deficiency of the left lower extremity in Mobius Syndrome (anterior view).

**Figure 2:**
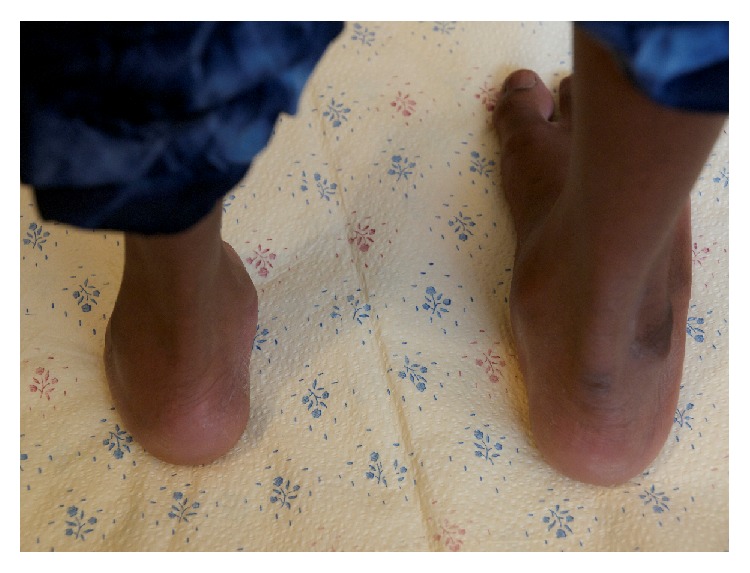
View from posterior of transverse deficiency.

**Figure 3:**
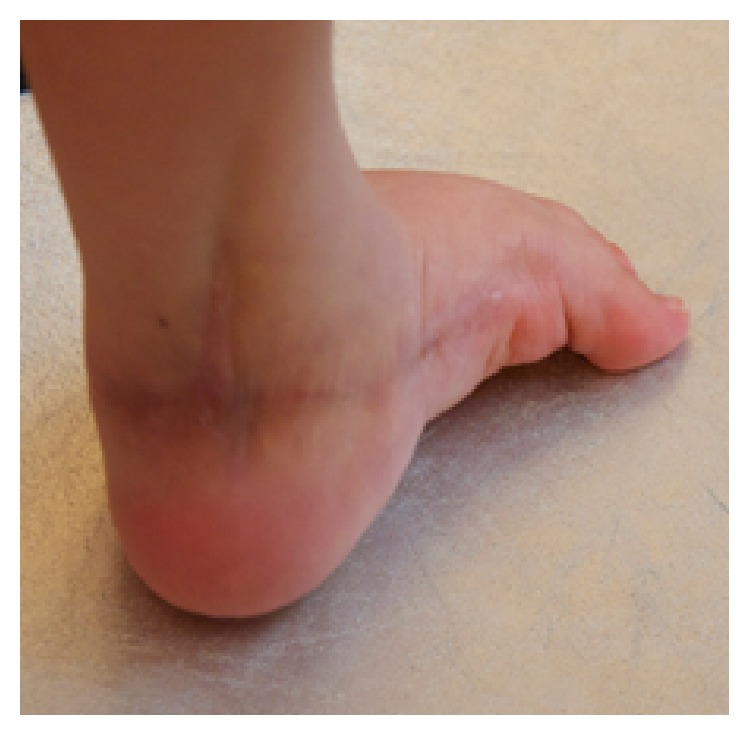
Clubfoot status after revision surgery with recurrent deformity, posterior view.

**Figure 4:**
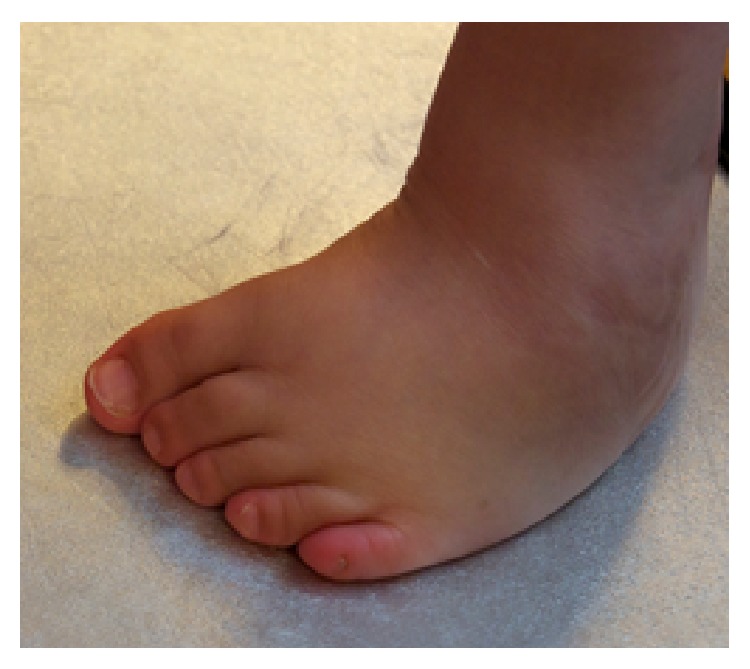
Clubfoot status after revision surgery with recurrent deformity, anterior view.

Twenty-three individuals with Mobius Syndrome were examined at the Mobius Syndrome Foundation meeting in July 2014. Age range was 6 months to 64 years, with 15 females and 8 males. Data is summarized in Tables 1 and 2. Fourteen individuals had clubfoot, 10 of which were bilateral. All underwent initial treatment with manipulation and casting; 8 subsequently required at least one surgical procedure for correction. Seven individuals required revision surgical treatment; one of the seven surgeries was split anterior tendon transfer to balance the muscle forces on the foot. This surgery is often required after nonoperative clubfoot treatment. Two of the four unilateral cases required surgical treatment. No revisions were needed. Figures 1 and 2 show a transverse deficiency at the level of the left ankle in an individual with Mobius Syndrome. Figures 3 and 4 demonstrate residual clubfoot in a patient who had already undergone primary and revision surgery for clubfoot correction.

Four cases of Poland's Syndrome were identified based on absence of the pectoralis tendon in the axillary fold. One had no hand abnormality, one had a smaller hand compared to the contralateral side, one had a distal transverse deficiency of the small finger, and one had syndactyly status after release. All were on the right side. For Poland Syndrome, one was between the thumb and index finger and responded well to release. The second was a bilateral polysynactyly that had undergone multiple release at the age of 8 months and subsequently recurred.

Five individuals had abnormal Adams forward bending tests, which is a sensitive test for scoliosis. The presence of asymmetry on this test is indicative of possible scoliosis and is used in the screening exam for scoliosis [23]. Two of these individuals had a previous X-ray diagnosis of scoliosis; however, only one was able to bring imaging. Imaging demonstrated a 28-degree right thoracic curve and a 16-degree left lumbar curve. The remaining three individuals with an abnormal Adams test had not undergone X-ray examination of their spines.

All families of children reported “low tone” during the neonatal period, and the majority had delayed motor milestones with ambulation at approximately 2 years. One individual had bilateral hip dysplasia successfully treated with Pavlik Harness.

## 4. Survey Results

One hundred and forty-six patients initiated the survey from May 11, 2013, to January 22, 2014. One hundred and nine completed at least some of the questions, although only ninety-six of these patients fully completed the survey. Only the fully completed surveys from the one ninety-six respondents are included in the analysis. Data is summarized in Table 3.

Regarding the upper extremity, there were many reported differences. Thirty-five had abnormal, missing, or shortened fingers (36.46%). Seventeen had syndactyly (17.71%). Sixteen had stiffness in the arm or shoulder (16.67%). Twenty-eight had nerve deficits in the arms (29.27%). Three had other diagnoses relevant to the upper extremity that were not asked about in the survey (3.125%).

Nearly half of individuals reported a difference in the lower extremity. Twenty-nine reported stiffness in the lower extremity (30.28%). Seventeen had abnormal toes (17.71%). Six had fused toes (6.250%). Forty had been diagnosed with clubfoot (41.67%). Three had been diagnosed with congenital vertical talus (3.125%). Thirty-seven have flat feet (38.54%). Twenty-three had nerve deficits in the legs (23.96%). Nine of the ninety-six had other diagnoses relevant to the lower extremity that were not addressed (9.375%). Seven patients specifically mentioned problems with weak or underdeveloped calves in their responses. Deformity may be severe and not necessarily symmetric. Despite modern treatment methods that have had increased success rates relative to historical primary surgical treatment, many individuals required surgical care and revision surgical procedures for clubfoot in our series.

Twenty-nine of the ninety-six patients had been diagnosed with missing or weak chest muscles (30.21%). Fourteen had been diagnosed with missing or weak back muscles (14.58%). Twenty-three had an atypical contour of the sternum, a sunken chest, pigeon chest, or asymmetry (23.96%). Twenty-five had a shoulder or scapula abnormality (26.04%). Eight had a diagnosis of the trunk other than those addressed (8.333%).

Twenty-seven of the ninety-six respondents had a diagnosis of scoliosis (28.13%). Ten had a diagnosis of kyphosis (10.42%). Five had a diagnosis of lordosis (5.208%). Two had a diagnosis of missing or malformed bones in the spine (2.083%). None of the patients said they had a diagnosis of fused bones in the spine. Four had spinal abnormalities that were not addressed in the survey (4.167%).

Eighty-five of ninety-six patients cannot move their eyes side to side (88.54%). Ninety are not able to smile/raise their eyebrows (93.75%). Seventy-four had a small jaw bone (77.08%). Thirteen of the ninety-six patients had been diagnosed with a cleft palate (13.54%), and seventeen had a high palate (17.71%). Thirty-nine had facial deformities that were not addressed in the survey (40.63%).

Twenty-five of the ninety-six were identified as having problems with anesthesia (26.04%). Unfortunately, we were unable to detail the specifics of trouble with anesthesia in this series. Future research efforts will be focused in this area.

Ninety-four of the patients responded to questions about family members with Mobius Syndrome. Five of them reported family members with Mobius Syndrome (5.319%). Another five were unsure about Mobius Syndrome in the family because of issues with adoption.

## 5. Discussion

Mobius Syndrome is a rare congenital disorder with a significant risk of associated orthopedic pathology. While the etiology of Mobius Syndrome has yet to be definitively elucidated, numerous promising theories have been reported which may explain the high association with orthopedic pathology [1, 7, 9–11]. Our study aimed to directly identify orthopedic pathology in a cohort of individuals with Mobius Syndrome. The most common associations were clubfoot deformity (42%), planovalgus foot (38%), and upper extremity digital malformation (37%). 45% of patients had some involvement of their spine as well. Perhaps most importantly for surgeons, more than a quarter of the patients surveyed had anesthetic difficulties. The most significant weakness of this study is that it does not include direct patient evaluation for the patients surveyed, and patients self-reported anatomic differences are evaluated. While this introduces the possibility of misdiagnosis, individuals were directed to report only pathology that has been diagnosed by a physician. A relative strength of this approach is that its patients are not filtered by subspecialty evaluation. Previous reports that included orthopedic pathology in a review were presented by facial surgeons, which might generate bias and skew the series as patients self-select for surgical consideration. One previous study identified an 86% overall incidence of extremity malformation, with 61 percent of patients having an upper extremity difference and 69 percent having a lower extremity difference [1].

A retrospective review in the plastic surgery literature including 27 patients reported 5 patients with scoliosis (18.52%) and 10 patients with lower limb deformity including clubfoot (2, 7.407%), flatfoot (1, 3.704%), metatarsus adductus (1), valgus (2), peroneal nerve deficiency (1), and congenital amputation (2). Three patients had syndactyly of the toes [24]. Upper extremity differences were also reported and included missing digits (4), syndactyly (1), radial aplasia (1), short humerus (1), hypoplastic radius/ulna (1), brachydactyly (1), and clinodactyly (1) [24]. Our study reports a higher incidence of clubfoot, flatfoot, and scoliosis, which may be due to decreased likelihood of recognition/reporting of these conditions in the craniofacial setting.

Due to our study design, we are unable to confirm the patients reported pathology with physical exam in all cases; however, the findings of the series of patients were similar to the overall survey with a slightly higher percentage of clubfoot and a slightly lower percentage of upper extremity syndactyly and scoliosis in the patient series. Our study's greatest strength is the high number of responses recorded and analyzed. This was backed up with a series of 23 patients demonstrating similar incidence. Mobius Syndrome appears to have a remarkably high association with musculoskeletal pathologies, many of which require treatment at an early age for optimal outcomes (clubfoot, hip dysplasia).

Parents of children with Mobius Syndrome and bilateral clubfoot should be counseled that their child's clubfoot may be more likely to require surgical or revision surgical care than idiopathic clubfoot. Unilateral deformity appears to be less severe and more responsive to standard treatment.

Perhaps our most important conclusion from a surgical standpoint is the high incidence of perceived anesthetic complications on the part of our cohort. As surgeons strive to first “do no harm,” we should have our patients with Mobius Syndrome carefully evaluated for anticipated airway difficulty and anesthetized by only those with significant experience with challenging airways and potential anesthetic complications.

In summary, this series of patients with Mobius Syndrome represents the largest series of patients to date establishing the incidence of orthopedic manifestations in the syndrome. It is our hope that this information will be helpful for those physicians who encounter this rare disease, in terms of identifying and providing timely treatment for associated conditions and for assisting in counseling patients and their families regarding treatment of their condition.

## Figures and Tables

**Table 1:** Patient series results.

Patient	Age	M/F	Clubfoot	Surg.	Revision/#	Foot, other	Pes planovalgus	Transverse deficiency	Hip dysplasia	Hip Surg.
1	16	F	R	R						
2	7	F	B	B	Bx1					
3	8	M						L (ankle)		
4	2	F								
5	64	F								
6	60	F	L							
7	11	F	B							
8	7	M	B	Y	Bx1					
9	27	F								
10	6	M	B	B	Bx2					
11	2	F	B	B	B Splatt					
12	0.5	F								
13	3	M	L	L	Pending					
14	1	M								
15	22	F	B	B	Bx1					
16	2.6	F	B							
17	1	M	R						Y	N
18	19	F	B	B	Lx2					
19	13	F				Small 5th toe	B			
20	3	M	B							
21	2	F				Absent P2 1st ray				
22	1	M	B						Y	N
23	41	F								
%			60.9%	34.8%	30.4%	8.7%	4.3%	4.3%	8.7%	8.7%

Age (years), M/F: male/female, Surg.: surgical treatment, B: bilateral, R: right, L: left, x-#: number of revisions, and Hip Surg.: hip surgery.

**Table 2:** Patient series contiued.

Patient	Age	M/F	Scoli. (xr)	Adams	Poland's	Hand	Side	Surg.	Revision/#
1	16	F		R thoracic					
2	7	F				1-2 cases of webspace syndactyly	R	R	
3	8	M				Polysyndactyly	B	B	Pending
4	2	F							
5	64	F							
6	60	F							
7	11	F							
8	7	M	Y	R thoracic					
9	27	F	Y	R thoracic, L lumbar					
10	6	N			R				
11	2	F							
12	0.5	F							
13	3	M							
14	1	M							
15	22	F		R thoracic					
16	2.6	F			R	Small hand			
17	1	M							
18	19	F	Y	R thoracic/L lumbar					
19	13	F				Small 5th finger w/o nail	B		
20	3	M							
21	2	F							
22	1	M			R	Small hand	R		
23	41	F		R thoracic	R	Syndactyly s/p release	R		
%			13.0%	26.1%	17.4%	26.1%	21.7%	8.7%	4.3%

Age (years), M/F: male/female, Scoli. (xr): scoliosis confirmed by radiography, R: right, L: left, and B: bilateral.

**Table 3:** Patient survey results.

	Number	Percent
Difference in digital anatomy	35	36.5%
Syndactyly	17	17.7%
Upper extremity nerve deficit	28	29.2%

Difference in digital anatomy	17	17.7%
Syndactyly	6	6.3%
Clubfoot	40	41.7%
Congenital vertical talus	3	3.1%
Planovalgus foot	37	38.5%

Missing/weak pectoralis	29	30.2%
Missing/weak back musculature	14	14.6%
Sternal abnormality	23	24.0%

Scoliosis	27	28.1%
Kyphosis	10	10.4%
Lordosis	5	5.2%
Missing bone in spine	2	2.1%

Difficulty with anesthesia	26	27.1%

Positive family history	5	5.2%
